# A novel UHMWPE anchor for improving the pullout resistance of pedicle screws

**DOI:** 10.3389/fbioe.2026.1741050

**Published:** 2026-01-14

**Authors:** Che-Kuang Lin, Fu-Shan Jaw, Tai-Horng Young

**Affiliations:** 1 Department of Biomedical Engineering, College of Medicine and College of Engineering, National Taiwan University, Taipei, Taiwan; 2 Division of Neurosurgery, Department of Surgery, Far Eastern Memorial Hospital, New Taipei, Taiwan

**Keywords:** anchor, bone density, pedicle screw, pilot hole, pullout

## Abstract

**Introduction:**

The ability to maintain bone purchase and resist pullout is considered a basic requirement for pedicle screws. However, poor bone density or oversized pilot holes can increase the risk of screw loosening and pullout. Our team developed a novel UHMWPE anchor that attaches to the proximal portion of the pedicle screw to improve engagement with the bone, even in conditions with poor bone quality or large pilot holes.

**Methods:**

Synthetic bone blocks simulating normal bone density (15 PCF) and osteoporotic bone (10 PCF) were used to investigate the pullout strength of anchored pedicle screws and traditional pedicle screws. Five screw constructs were evaluated: (A) 4.0 mm pedicle screw and 3.0 mm pilot hole; (B) 4.0 mm pedicle screw with a 4.0 mm anchor and 3.0 mm pilot hole; (C) 4.0 mm pedicle screw with a 4.0 mm anchor and 3.2 mm pilot hole; (D) 4.0 mm pedicle screw and 3.2 mm pilot hole; (E) 5.0 mm pedicle screw and 3.2 mm pilot hole.

**Results:**

The anchor-based 4.0 mm screw with a 3.0 mm pilot hole (group B) had the highest pullout strength among all groups, both in normal bone and osteoporotic bone. Even when using a larger 3.2 mm pilot hole, the pullout strength of the anchored 4.0 mm screw was within 90% of the values recorded with the 3.0 mm pilot hole. Notably, the average pullout strength of the 4.0 mm pedicle screw with anchor was higher than the 5.0 mm pedicle screw without an anchor in both bone densities.

**Conclusion:**

The results of this study demonstrate that the novel anchor can significantly increase the pullout strength of pedicle screws. The anchor also allows for more flexibility when selecting a screw size, especially when the insertion space is limited.

## 1 Introduction

Pedicle screw fixation is considered the golden standard for contemporary spinal stabilization, yet screw loosening commonly results in construct failure and revision surgery (Hsieh et al., 2022; Arena et al., 2024; Li et al., 2024). Loosening is especially problematic in elderly or osteoporotic patients, where weak or low density bone compromises the integrity of the screw–bone interface and undermines long-term stability (Bokov et al., 2021; Narayanan et al., 2024). Even in stronger bone, a misdirected screw that threatens neural or vascular structures often must be withdrawn and reinserted along a new trajectory, which enlarges the original pilot hole and further reduces purchase.

Conventional rescue strategies, such as using a larger diameter screw, packing the tract with bone chips, or injecting polymethyl-methacrylate (PMMA) cement, each carry well-recognized limitations (Gong et al., 2022; Hsieh et al., 2022). Larger screws risk fracturing the fragile pedicles and reduce the volume of intact bone by requiring a larger screw hole. PMMA improves initial fixation but introduces risks of thermal necrosis, embolic leakage, and permanent foreign-body retention (Gong et al., 2022; Takahashi et al., 2024). Bone-graft packing yields variable, biology-dependent results and does not deliver immediate mechanical stability.

While expandable and fenestrated cement-augmented screws have been developed to address these shortcomings, they also introduce proprietary hardware, increased cost and complexity, and can complicate future implant removal (Miyamoto et al., 2001; Gazzeri et al., 2020; Saadeh et al., 2020; Kwak et al., 2022). Consequently, there remains a need for a simple, reversible, and readily available method to restore screw stability after pilot-hole enlargement without increasing the screw diameter or relying on cement. A finite element study by Prajapati AK et al. indicated that, in addition to bone density, the pull-out strength of pedicle screws can be enhanced through design modifications, particularly by optimizing proximal and distal thread parameters using finite element analysis and design of experiments approaches during primary insertion (Prajapati et al., 2024). However, the effect of thread parameters on different pilot hole sizes or in revision cases is still unclear. We therefore developed a novel anchor-based screw system that functions as a detachable polymer sleeve seated at the screw entry point. As the screw is advanced, the ultra-high-molecular-weight polyethylene (UHMWPE) anchor expands radially, re-establishing intimate contact between the screw thread and surrounding bone. The concept is similar to interference-fit dowels in carpentry, where the holding strength is restored by filling the void rather than enlarging the fastener itself. Because the anchor is an accessory rather than an integral part of the screw, the system does not require a special screw design or novel instrumentation. Preliminary bench work suggested that the anchor generates a post-peak “load plateau,” maintaining pullout resistance after initial yielding and mitigating the abrupt loss of purchase typical of stripped screws. This study aimed to investigate how the detachable UHMWPE anchor affected the axial pullout strength of pedicle screws in comparison to standard pedicle screws inserted in normal-density and osteoporotic bone models. We hypothesized that the pullout strength of anchor-augmented pedicle screws with smaller diameter would outperform screws with a larger diameter alone.

## 2 Methods

### 2.1 Testing setup

**FIGURE 1:**
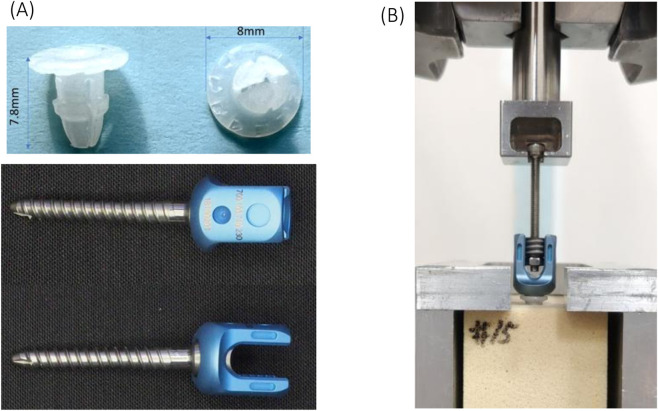
**(A)** Novel UHMWPE anchor and commercial pedicle screws used in this study; **(B)** Pullout testing setup.

The axial pullout strength was assessed through mechanical testing in accordance with ASTM F543-17 (ASTM international, 2017), with the screws inserted into rigid polyurethane foam (Sawbones®, Pacific Research Labs, Vashon, WA, United States) manufactured to ASTM F1839 tolerances. Two bone densities were simulated; 15 PCF (normal density, catalog #1522-02) and 10 PCF (osteoporotic, catalog #1522-01). Five separate groups were established, with 5 samples tested in each group: (A) 4.0 mm pedicle screw and 3.0 mm pilot hole; (B) 4.0 mm pedicle screw plus 4.0 mm anchor and 3.0 mm pilot hole; (C) 4.0 mm pedicle screw plus 4.0 mm anchor and 3.2 mm pilot hole; (D) 4.0 mm pedicle screw and 3.2 mm pilot hole; (E) 5.0 mm pedicle screw and 3.2 mm pilot hole (Figure 1A). Pilot holes were drilled perpendicular to the block surface with calibrated twist drills held in a vertical collet to a depth of 30 mm, controlled with a custom stop collar. Prior to testing, all bone blocks were visually inspected for voids or machining defects.

### 2.2 Implant constructs

Commercial 4.0 mm (inner shaft diameter 3.0 mm) and 5.0 mm (inner shaft diameter 3.2 mm) diameter pedicle screws 30 mm in length (OCTOPODA system, Bricon Medical, German) (Figure 1B) served as the base screws in our study. Anchors were machined from ultra-high-molecular-weight polyethylene (UHMWPE) chosen for its low friction coefficient, high impact resistance, and proven biocompatibility. The anchor geometry (cylindrical sleeve with peripheral expansion fins) (Figure 1B) was designed to achieve an interference fit when seated in the pilot-hole. Immediately before screw insertion, each anchor was advanced by hand until the underside of the anchor head was flush with the block surface.

### 2.3 Biomechanical testing protocol

All tests were executed on a servo-hydraulic universal testing machine (MTS Criterion C42.503; Eden Prairie, MN, United States). Specimens were clamped in a self-aligning vice to ensure axial loading. Screws were inserted with a digital torque driver to 1.0 Nm to standardize seating torque. Axial pullout was performed at a constant cross-head displacement rate of 5 mm/min until a 30% drop from peak load was detected, with the peak load (N) being recorded as the ultimate pullout strength. Real-time force–displacement data was sampled at 100 Hz.

### 2.4 Statistics

The pullout strength was recorded for the five different screw configurations (n = 5 in each group) in the two block densities (15 PCF and 10 PCF). A one-way ANOVA was conducted to evaluate whether there were any significant differences in pullout strength among the five groups in both block densities. The pullout strength of each screw configuration (A–E) was also compared directly between 15 PCF and 10 PCF blocks by independent t-test analysis.

## 3 Results

### 3.1 Normal-density bone (15 PCF polyurethane blocks)

**FIGURE 2:**
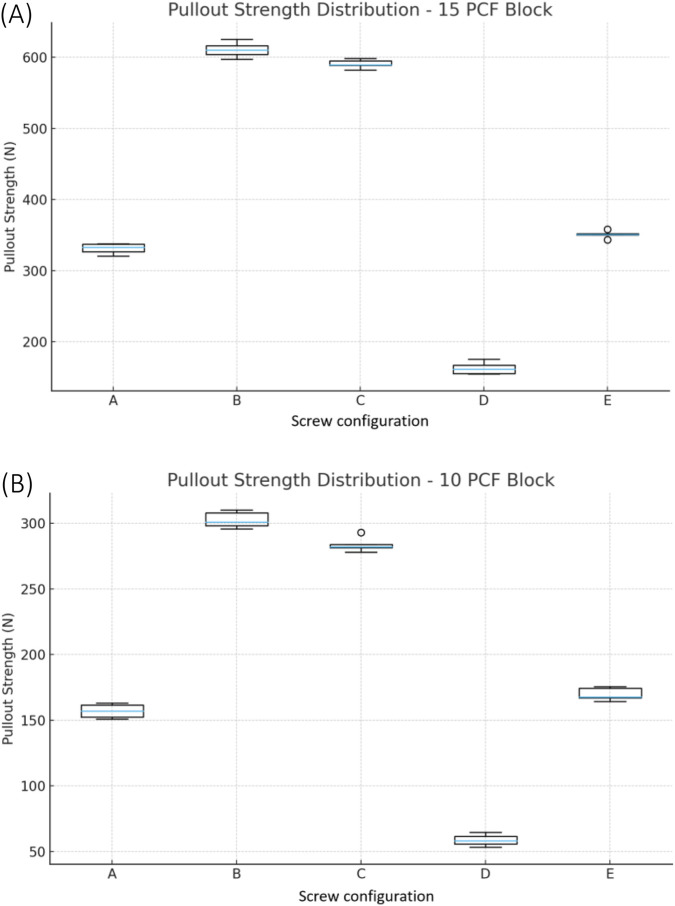
**(A)** Distribution of pullout strength across five screw configurations in the 15 PCF block; **(B)** Distribution of pullout strength across five screw configurations in the 10 PCF block.

In the normal-density bone (15 PCF foam), the 4.0 mm pedicle screw with 4.0 mm anchor inserted in a 3.0 mm pilot hole (group B) had a significantly higher pullout strength than the conventional screw-only configuration (both 4.0 mm (Group A) and 5.0 mm (Group E) pedicle screws, p < 0.01). Group B exhibited the greatest average pullout strength at 610.57 ± 10.78 N, followed by the group C at 590.76 ± 5.49 N. The screw-only group A produced the lowest average pullout strength of 331.16 ± 6.57 N (Table 1; Figure 2A).

**TABLE 1:** Comparison of the mean maximal pullout strength for five groups.

Normal-density bone (15 PCF polyurethane blocks)								
​	Group	Uint: N (Newton)						
		1	2	3	4	5	Average	S.D.
A	4.0 mm pedicle screw and 3.0 mm pilot hole	320.76	332.78	337.67	337.77	326.79	331.16	6.57
B	4.0 mm pedicle screw plus 4.0 mm anchor and 3.0 mm pilot hole	616.45	609.95	603.99	625.13	597.35	610.57	10.78
C	4.0 mm pedicle screw plus 4.0 mm anchor and 3.2 mm pilot hole	582.18	594.68	589.57	589.02	598.34	590.76	5.49
D	4.0 mm pedicle screw and 3.2 mm pilot hole	154.93	167.39	175.59	155.74	161.73	163.08	7.71
E	5.0 mm pedicle screw and 3.2 mm pilot hole	350.38	350.73	351.96	343.36	358.09	350.90	5.24

### 3.2 Osteoporotic bone (10 PCF polyurethane blocks)

In low-density osteoporotic bone (10 PCF foam), group B continued to outperform the conventional screw. Group B achieved an average pullout strength of 302.57 ± 6.53 N, compared to 156.53 ± 4.86 N and 169.73 ± 4.94 N for the 4.0 mm and 5.0 mm screw-only groups (group A and E), respectively. In osteoporotic bone, the pullout strength of group C was significantly lower than group B (P < 0.05) (Table 2; Figure 2B).

**TABLE 2:** Comparison of the mean maximal pullout strength for five groups.

Osteoporotic bone (10 PCF polyurethane blocks)								
​	Group	Uint: N (Newton)						
		1	2	3	4	5	Average	S.D.
A	4.0 mm pedicle screw and 3.0 mm pilot hole	150.76	152.34	156.97	161.46	163.14	156.93	4.86
B	4.0 mm pedicle screw plus 4.0 mm anchor and 3.0 mm pilot hole	310.08	298.26	300.79	308.03	295.69	302.57	6.23
C	4.0 mm pedicle screw plus 4.0 mm anchor and 3.2 mm pilot hole	283.87	277.87	281.96	281.20	292.85	283.55	5.04
D	4.0 mm pedicle screw and 3.2 mm pilot hole	64.47	58.24	55.72	61.56	53.25	58.65	4.01
E	5.0 mm pedicle screw and 3.2 mm pilot hole	167.13	164.14	167.53	174.39	175.47	169.73	4.94

### 3.3 Statistical analysis

For the 15 PCF block, the one-way ANOVA showed a highly significant difference between the groups, with an F-statistic of 2,897.07 and a p-value less than 0.0001. Similarly, for the 10 PCF block, the F-statistic was 1733.41 with a p-value of less than 0.0001, indicating significant differences between the groups (Table 3).

**TABLE 3:** One-way ANOVA for pullout strength.

Block density	F-statistic	p-value
15 PCF	2,897.07	<2.53e-27
10 PCF	1733.41	<4.25e-25

Each screw configuration (A–E) was also compared directly between the 15 PCF and 10 PCF blocks. Independent t-tests revealed a significantly higher pullout strength in 15 PCF across all groups (p < 0.001 for each comparison). The effect sizes (Cohen’s d) were extremely large, confirming block density as a dominant factor influencing pullout performance (Table 4).

**TABLE 4:** Mean pullout strength for each screw configuration (A–E) is shown for both 15 PCF and 10 PCF blocks. Across all groups, pullout strength was consistently higher in 15 PCF compared to 10 PCF blocks.

Group	Mean 15 PCF (N)	Mean 10 PCF (N)	Test used	p-value	Cohen’s d
A	331.15	156.93	t-test	<0.001	26.96
B	610.57	302.57	t-test	<0.001	34.98
C	590.76	283.55	t-test	<0.001	52.13
D	163.08	58.65	t-test	<0.001	15.20
E	350.90	169.73	t-test	<0.001	35.58

## 4 Discussion

Pedicle screw pull-out strength is influenced by multiple factors, including bone density, screw design, and insertion-related variables such as insertion depth, all of which may contribute to compromised fixation (Salunke et al., 2023; Prajapati et al., 2024). Screw loosening and the subsequent need to reposition pedicle screws is a common challenge in spinal surgery, particularly in osteoporotic bone (Rometsch et al., 2020; Oberthür et al., 2024). When a screw’s initial placement is suboptimal – for example, breaching a pedicle or threatening nearby neural structures – surgeons often must remove and reinsert the screw along a new trajectory. Traditionally, a compromised pilot hole was managed by either using a larger-diameter screw or augmenting the hole with bone cement, but both approaches carry considerable drawbacks. Enlarging the screw size can risk fracturing the pedicle or further damaging porous bone, and it may not be feasible if appropriately sized hardware is unavailable (Matsukawa et al., 2021; Schömig et al., 2023). Cement augmentation can improve initial fixation, but it prolongs the surgery time and introduces other post-surgical risks like thermal bone injury and cement leakage (Lai et al., 2011; Gazzeri et al., 2020; Kim et al., 2020; Boucas et al., 2023).

In a mechanical study, Varghese et al. (Lai et al., 2011; Gazzeri et al., 2020; Kim et al., 2020; Boucas et al., 2023) indicated that the pullout strength of pedicle screws decreases with a reduction in bone density. Similarly, our study found that the pullout strength in each group was significantly lower in osteoporotic bone than in normal-density bone, with a decrease of 52.61% in group A, 50.44% in group B, 64.04% in group C, 52.00% in group D, and 51.63% in group E. The group with 3.2 mm pilot hole and 4.0 mm screw (group D) showed the greatest difference in pullout strength between normal bone and osteoporotic bone. Regardless of the bone density, group B (4.0 mm pedicle screw plus 4.0 mm anchor and 3.0 mm pilot hole) had the highest pullout strength among all groups. Using an anchor with an oversized 4 mm screw resulted in a pullout strength that was 174% higher in normal-density bone and 206.74% higher in osteoporotic bone compared to the model that used a larger 5.0 mm diameter screw (group E). When an anchor was used to support the 4.0 mm screw in osteoporotic bone, the average pullout strength was close to the values for a 4.0 mm screw alone in normal-density bone (302.57 N versus 331.16 N).

In enlarged pilot hole scenarios simulating clinical revisions, the anchor mechanism preserved and even improved fixation stability. These results highlight the potential clinical advantages of the anchor-based screw which allows surgeons to salvage a loosened screw and maintain strong fixation without resorting to larger implants or cement. With the 3.2 mm pilot hole, the pullout strength of the anchor-based 4.0 mm screw (group C) was still higher than the 4.0 mm or 5.0 mm screw alone (group A and group E). Regardless of the bone density, the results for the 4.0 mm anchored screw with 3.0 mm pilot hole (group B) were similar to the 3.2 mm pilot hole (group C), which suggests that anchor expansion may alter load distribution along the screw–anchor–bone interface, increase radial compression of the surrounding material, and enhance friction between the anchor and adjacent structures, thereby mitigating the adverse effects of an enlarged pilot hole.

**FIGURE 3:**
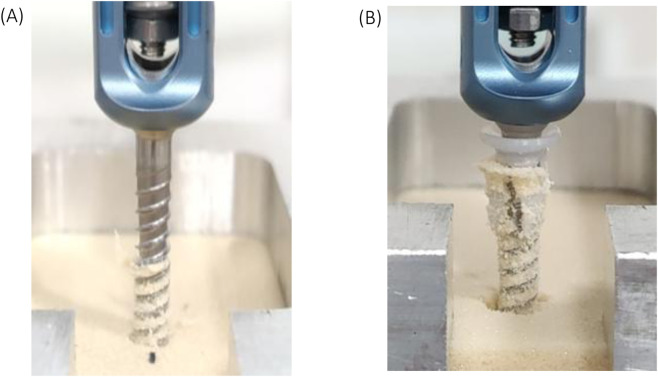
Compared with the screw-only configuration **(A)**, the screw with anchor caused more material to be removed from the insertion hole after pullout **(B)**.

The position of the anchor at the proximal end of the screw caused the test block to bulge and collapse at the moment of failure in the pullout test. As the screw is pulled from the block it strips the threaded hole, creating a hole larger than the screw diameter (Figure 3). As a result, the distal threads fail to fully engage as the proximal anchor loses purchase, which may explain why the anchor’s ability to maintain fixation plays a dominant role in the overall pullout strength. Previous studies have shown that pilot hole expansion significantly reduces the screw pullout strength (Lai et al., 2011; Gazzeri et al., 2020; Kim et al., 2020; Boucas et al., 2023). A pilot hole that exceeds the inner diameter of the screw reduces the contact surface area between the screw and hole, leading to a loss of pullout strength (Lai et al., 2011; Gazzeri et al., 2020; Kim et al., 2020; Boucas et al., 2023). This situation was observed in group D (3.2 mm pilot hole for 4.0 mm screw) where the pullout strength was approximately 50% lower than group A with a smaller 3.0 mm pilot hole. When faced with a stripped or enlarged pilot hole, surgeons have historically relied on a few strategies to regain screw purchase. Inserting a larger screw can sometimes improve grip, but using oversized hardware in fragile or osteoporotic bone can compromise the bone’s integrity. This practice may lead to microfractures or accelerate loosening over time. Moreover, larger screws are not always suitable due to anatomical constraints or limited availability of incrementally bigger implants (Matsukawa et al., 2016; Chang et al., 2021). Although the anchor increased the pull-out strength of the pedicle screw, vertical cracks were observed around the surrounding material during the pull-out process (Figure 3B). These cracks suggest localized stress concentration at the anchor–material interface, which may have induced microfracture of the synthetic bone structure and resulted in localized collapse near the screw hole during screw extraction. Therefore, reducing stress concentration at the anchor–bone interface represents an important objective for future design optimization. The use of a polymer anchor offers several practical benefits. **UHMWPE** is an inert, non-degradable material widely used in orthopedic implants, ensuring the anchor will not resorb over time and can withstand cyclic loads without cracking (Pokorný et al., 2024; Smith et al., 2024). It also does not produce significant imaging artifacts, which means postoperative imaging can still assess screw placement. Importantly, because the anchor is not affixed permanently to the bone, the screw-anchor assembly can be backed out if needed, much like a standard screw. This removability contrasts with cement augmentation or certain expandable screws, which are difficult to reverse once deployed. From a cost standpoint, manufacturing a small polymer insert is less expensive than designing specialized expandable screws or performing cement procedures. Accordingly, although UHMWPE does not facilitate osteointegration, its use as a sleeve material is considered acceptable in the context of posterior spinal fixation, which primarily serves as a temporary stabilization system until solid vertebral fusion—typically achieved within 3–6 months—reduces reliance on implant-bone interface mechanics. In this study, the anchor length was defined as 7.8 mm, corresponding to approximately one quarter of the screw length used in the experiment. This design approach is consistent with existing pedicle screw concepts, in which enhanced fixation features—such as denser thread geometry or increased core diameter—are incorporated in the proximal one-quarter to one-third of the screw shaft to strengthen fixation within the pedicle, where cortical bone density is relatively high (Weinstein et al., 1992). Another reason for limiting the sleeve application in this study to the proximal quarter of the screw was consideration of the location of the lumbar pedicle isthmus. Avoiding the use of full-length anchors helps prevent excessive stress concentration within the pedicle during screw insertion, which could otherwise compromise fixation strength or even result in pedicle fracture.

There are some limitations to this study. First, all biomechanical tests were performed in uniform-density polyurethane foam blocks with precisely drilled pilot holes. Although ASTM 1839-compliant foam is generally considered an accepted surrogate for cancellous bone and is routinely relied on for regulatory submissions of new screw designs, it cannot fully replicate the cortical shell, trabecular heterogeneity, or remodeling capacity of living vertebrae. In addition, in clinical practice, a screw may be advanced, backed out, and redirected multiple times, or the bone defect may be irregular or cystic. Consequently, real-world factors such as cyclic loading, partial fusion, progressive osteoporosis, and non-cylindrical insertion holes may still influence the anchor performance. Second, only the pullout strength of the screws was evaluated. While axial pullout is an acceptable standard for qualifying new bone screws under ASTM F543-17, additional modes such as torsion, bending, and fatigue would be beneficial for characterizing the behavior of the screws and anchor. Nevertheless, because the anchor is an accessory that leaves the core screw geometry unchanged, the pullout test detailed in ASTM F543-17 is sufficient, and no novel test paradigms were necessary. Third, this study focused on 4.0 mm–5.0 mm pedicle screws because their small diameter and relatively uniform thread geometry minimize confounders and enable standardized testing of the anchor mechanism. In practice, commercial pedicle screws are available with varying outer diameters, core diameters, thread depths, and tip designs, all of which may affect the pullout strength. Fourth, the long-term *in vivo* behavior of an ultra-high-molecular-weight polyethylene (UHMWPE) anchor remains unknown. While UHMWPE has an excellent orthopedic track record, it is susceptible to wear debris generation, polymer creep, or chronic interface motion. Finally, because experimental data on *in situ* anchor deployment within the pedicle remain limited, the optimal anchor size cannot yet be definitively determined based on the present prototype-based biomechanical results. However, these factors were beyond the scope of this short-term study but may be considered in future work. At the next stage, a one-level interbody fusion model will be established to simulate different bone densities by varying the Young’s modulus of cancellous bone, and finite element analysis will be used to evaluate stress distribution among the pedicle screw, sleeve, and surrounding bone tissue for further anchor optimization.

## 5 Conclusion

In conclusion, the novel UHMWPE anchor presented in this study was found to improve screw purchase and significantly increase the resistance to pullout in both normal and osteoporotic bone. This simple anchor mechanism may reduce the risk of pedicle screw loosening and pullout, which are particularly prevalent in osteoporotic bone and after screw revision.

## Data Availability

The raw data supporting the conclusions of this article will be made available by the authors, without undue reservation.
